# Determination of Hardness and Fracture Toughness of Y-TZP Manufactured by Digital Light Processing through the Indentation Technique

**DOI:** 10.1155/2021/6612840

**Published:** 2021-02-12

**Authors:** Ziyu Mei, Yuqing Lu, Yuxin Lou, Ping Yu, Manlin Sun, Xin Tan, Junjing Zhang, Li Yue, Haiyang Yu

**Affiliations:** State Key Laboratory of Oral Diseases, National Clinical Research Center for Oral Diseases, West China Hospital of Stomatology, Sichuan University, Chengdu 610041, China

## Abstract

**Objective:**

The purpose of the study was to determine the hardness and fracture toughness of dental yttria-stabilized tetragonal zirconia polycrystal (Y-TZP) manufactured by digital light processing (DLP) technology to study its clinical prospects.

**Methods:**

The experimental group was DLP-manufactured zirconia, and the control group was milled zirconia. The hardness was investigated under a range of test loads (0.49 N, 0.98 N, 1.96 N, 4.90 N, 9.81 N, 29.42 N, 49.03 N, 98.07 N, and 196.1 N). Meyer's law was applied to describe the indentation size effect (ISE). Meanwhile, the PSR model and MPSR model were utilized to generate true hardness values. The cracks were observed to be induced by indentation under loads above 49.03 N, while the cracks showed the radial-median type under the load of 196.1 N, under which the fracture toughness was calculated.

**Results:**

The true hardness of DLP-manufactured zirconia was 1189 HV based on the PSR model and 1193 HV based on the MPSR model, a bit lower than that of milled zirconia. The fracture toughness was 3.43 ± 0.29 MPa√m, which showed no statistical difference with the milled zirconia.

**Conclusion:**

The dental zirconia manufactured by the DLP 3D printing technique is similar to that manufactured by the conventional milling process in hardness and fracture toughness, thus having a promising future of clinical use.

## 1. Introduction

In recent years, yttria-stabilized tetragonal zirconia polycrystal (Y-TZP) is widely used in dentistry due to its excellent biocompatibility and mechanical properties, as well as its satisfying aesthetics. Usually, the zirconia prostheses are manufactured by the numerically controlled milling process, while there still exist some problems [1]. Firstly, the milling process is a subtractive operation from a presintered or fully sintered disc, which results in a massive waste of material. Secondly, the prostheses with fine structures such as deep fossae or grooves usually could not be shaped accurately, limited by the burs with certain diameters [2]. Thirdly, when milling the zirconia restorations with thin wall thickness, the process may cause mechanical damage due to the bur vibration [3].

With the rapid development of three-dimensional (3D) printing techniques, 3D printing ceramics becomes a hot issue in prosthodontics. This technique builds parts layer by layer with no need for machining or mould, thus possessing the characteristics of high production efficiency, high usage rate of material, and unlimited printing shapes [4]. Therefore, the 3D printing technique could be a potential alternative approach to manufacturing restorations with fine structures. Among the 3D printing techniques, the digital light processing (DLP) technique is relatively mature in ceramic forming. The DLP technique firstly forms green parts through light curing the resins in ceramic slurry [5]. Then, posttreatments involving debinding and final sintering are performed to obtain the dense ceramic parts. Compared to other 3D printing techniques, the DLP technique has advantages in forming small objects with high accuracy requirements, considered to be a preferred technique for 3D printing dental zirconia prostheses [6].

At present, there were a few studies on the mechanical properties of dental zirconia manufactured by 3D printing techniques. Lu et al. investigated that DLP-manufactured zirconia can achieve high flexural strength close to milled zirconia [7]. Li et al. printed the zirconia bridges and implants by stereolithography and displayed the defects in printed objects [6]. Osman et al. prepared zirconia implants based on the DLP technique, which achieved relatively high accuracy, surface quality, and flexural strength [8]. However, there is still a lack of studies on the hardness and fracture toughness of DLP-manufactured zirconia. Hardness is defined as the ability to resist plastic deformation, indicating the ease of surface polishing or scratching [9], which may have an impact on the aesthetic properties of zirconia prostheses. Fracture toughness describes the resistance ability to crack propagation under loading [9]. Thus, in this study, the dental zirconia manufactured by the DLP 3D printing technique was investigated and compared with milled zirconia to provide a further theoretical basis for its clinical use. The null hypothesis was that there was no statistical difference in the hardness and fracture toughness between DLP-manufactured zirconia and milled zirconia.

## 2. Materials and Methods

### 2.1. Specimen Preparation

All experiments were performed on zirconia discs with dimensions of diameter 20.0 mm and thickness 2.0 mm. The control group was Y-TZP, which was milled out of presintered Y-TZP discs (Zenostar, Ivoclar Vivadent, Liechtenstein) by a CAD/CAM machine (Wieland Zenostar mini, Ivoclar Vivadent, Liechtenstein) (MILL group). The experimental group was Y-TZP manufactured by a DLP stereolithography printing machine (Ceramatrix, QuickDemos Company, China) (DLP group), as is listed in Table 1. The zirconia slurry for printing was composed of 58 vol% Y-TZP powder and photocurable monomers. Firstly, the green parts of the DLP group were printed layer by layer under a light intensity of 90 mW/cm^2^ with 25 *μ*m each layer. Then, the debinding process was performed by putting the green parts into the debinding furnace under 100-450°C to remove organic parts. At last, the final sintering process was carried out on Y-TZP of the two groups by sintering them for 2 h at 1510°C with heating and cooling rates set at 300°C/h. All samples were observed under an optical microscope, and those without surface defects were selected for experiments. Before experiments, the samples were firstly ground with P400-P1200 SiC abrasive paper, followed by a polishing step with 9 *μ*m-1 *μ*m polishing slurry in a polishing machine (Struers, Copenhagen, Denmark).

### 2.2. Characterization of the Specimens

To characterize the specimens, the density, grain size, crystalline phase, and Young's modulus were investigated. The density was measured by means of Archimedes' method using the calculation

(1) $$\begin{array}{c} d=\frac{m_{1}\times \rho }{m_{3}-m_{2}}, \end{array}$$

where *d* is the density (g/cm^3^), *m*_1_ is the mass of the dried specimen in air (g), *m*_2_ is the mass of the water-impregnated specimen in water (g), *m*_3_ is the mass of the water-impregnated specimen (g), and *ρ* is the density of water which equals to 0.9982 g/cm^3^. The surface morphologies of Y-TZP were observed by a scanning electron microscope (SEM, Inspect F, USA). And the SEM images were used for analyzing the grain sizes using software ImageJ 1.52a (National Institutes of Health, USA). The crystalline phase structure was determined by an X-ray diffractometer (XRD, Empyrean, Netherlands), with a step size of 0.02° and a scan range of 10°-70°. Young's modulus was tested by an MHT^3^ microindentation tester. The maximum load was 10 N, and the holding time was 10 s. Young's modulus of each group was calculated by Indentation Software.

### 2.3. Hardness Measurements

According to ASTM C1327-2015 [10], the Vickers hardness was measured using a hardness tester (Zuanshi, China) under the following loads: 0.49 N (HV0.05), 0.98 N (HV0.1), 1.96 N (HV0.2), 4.90 N (HV0.5), 9.81 N (HV1), 29.42 N (HV3), 49.03 N (HV5), 98.07 N (HV10), and 196.1 N (HV20). There were 3 specimens for each group under each load, and 10 indentations were randomly made on each specimen with a dwelling time of 15 s. The Vickers hardness was calculated by the following expression:

(2) $$\begin{array}{c} \text{HV}=\frac{\alpha F}{d^{2}}, \end{array}$$

where HV is the Vickers hardness (HVn), *F* is the applied load (N), *d* is the mean value of the indentation diagonals (mm), and *α* is the indenter's geometrical constant, which equals 0.1891.

### 2.4. Fracture Toughness Measurements

The fracture toughness was measured using the indentation fracture method. Based on the Vickers hardness tests above, the length of the crack induced by the indentation *c* (mm) and half of the indentation diagonal *a* (mm) were measured to calculate the *c*/*a* ratio. The crack type could thus be confirmed [11]. When *c*/*a* > 2.5, the crack shows the Palmqvist type, and when *c*/*a* < 2.5, the crack shows the radial-median crack type. The fracture toughness values were then calculated using the correct equation for the corresponding crack type.

## 3. Result

### 3.1. Microstructural Characterization

**Figure 1:**
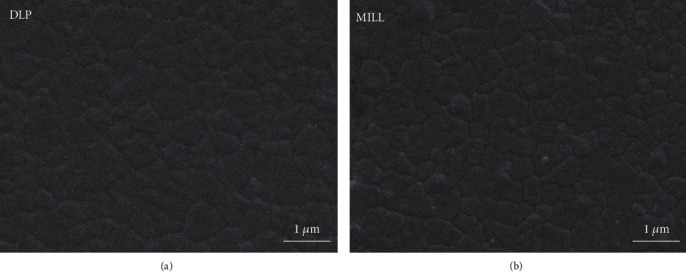
SEM images of the zirconia surface. (a) DLP group. (b) MILL group.

**Figure 2:**
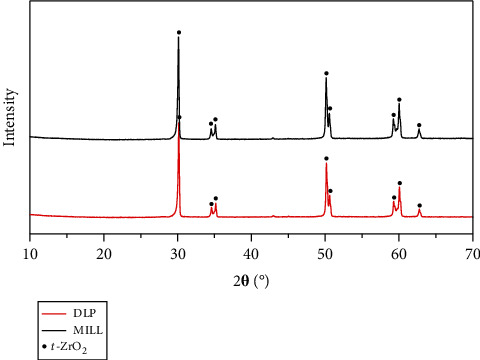
XRD patterns of zirconia.

As is shown in Table 2, there was no statistical difference between the two groups in density and grain size. The grains of both groups were of tight arrangement with uniform size (see Figure 1). The XRD pattern in Figure 2 shows that only the tetragonal phase was found in the two groups of zirconia. Young's modulus was 221.4 ± 2.2 GPa and 225.1 ± 3.4 GPa separately for the DLP group and the MILL group.

### 3.2. Apparent Hardness

**Figure 3:**
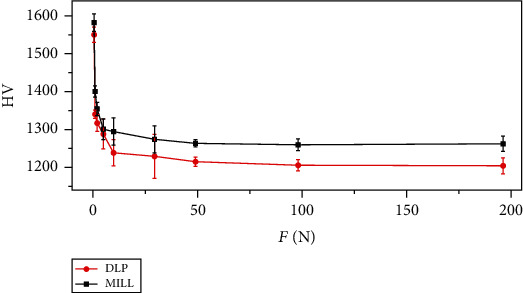
Plot of variations in hardness versus applied load. The error bars represent the standard deviation of hardness values of 30 indentations under each applied load.

**Figure 4:**
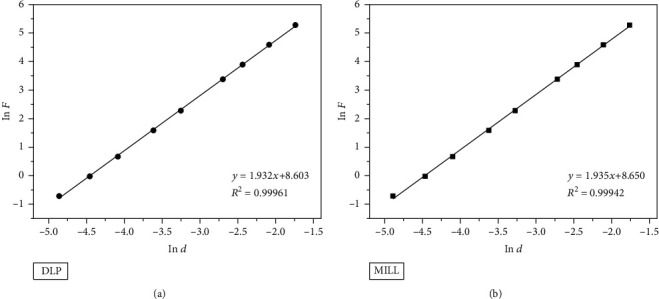
Fitting curves of ln *F* versus ln *d* based on Meyer's law. (a) DLP group. (b) MILL group.

The average hardness values versus applied indentation loads are plotted in Figure 3. It is observed that the hardness value HV decreased with the increasing indentation load *F*, which is called the normal indentation size effect (ISE) [12]. To verify the ISE, Meyer's law was applied [13]:

(3) $$\begin{array}{c} F=K∙d^{n}, \end{array}$$

where *F* is the load (N), *K* is a hardness constant, and *d* is the mean value of the indentation diagonal (mm). A linear regression analysis was conducted on ln *F* versus ln *d* to obtain the slope that represents Meyer's index *n*. The fitting curves are displayed in Figure 4. As is given in Table 3, Meyer's index was 1.935 ± 0.011 for the DLP group and was 1.947 ± 0.011 for the MILL group, both of which were smaller than 2, further confirming the normal ISE behavior of zirconia. Therefore, the obtained hardness values were apparent hardness dependent on the applied load; thus, two more empirical models were used to determine the true hardness.

### 3.3. True Hardness

**Figure 5:**
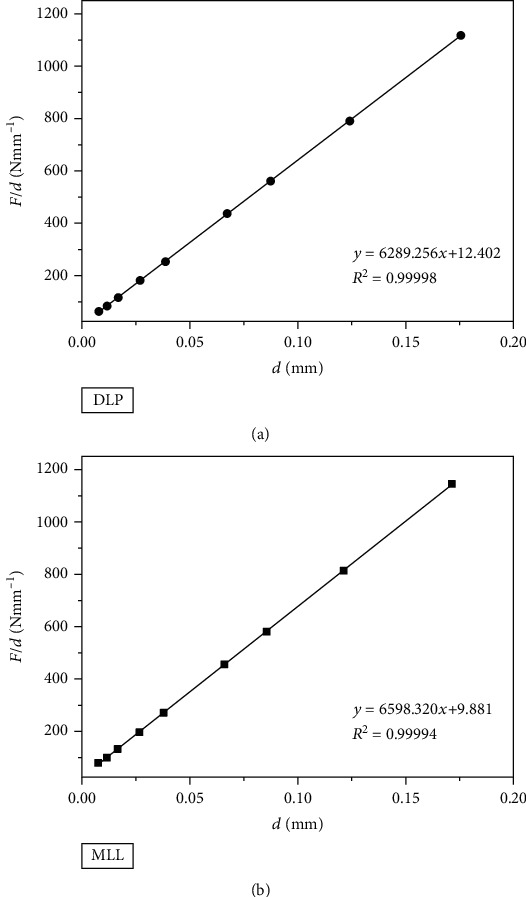
Fitting curves of *F*/*d* versus *d* based on the PSR model. (a) DLP group. (b) MILL group.

The proportional specimen resistance (PSR) model and the modified proportional specimen resistance (MPSR) model were used to determine the true hardness. The PSR model is a modification of Meyer's law [14], and it explains the relation between the load and the indentation diagonal. The expression is as follows:

(4) $$\begin{array}{c} F=a_{1}∙d+a_{2}∙d^{2}, \end{array}$$

where *a*_1_ (N mm^−1^) is a constant related to elasticity and *a*_2_ (N mm^−2^) is a constant related to plasticity. These two values were calculated by linear regression carried out on *F*/*d* versus *d* (see Figure 5). The results are presented in Table 4.

The MPSR model is developed based on the PSR model [15, 16], which adds a coefficient *a*_0_ (N) related to the material characterization and surface treatment:

(5) $$\begin{array}{c} F=a_{0}+a_{1}∙d+a_{2}∙d^{2}. \end{array}$$

**Figure 6:**
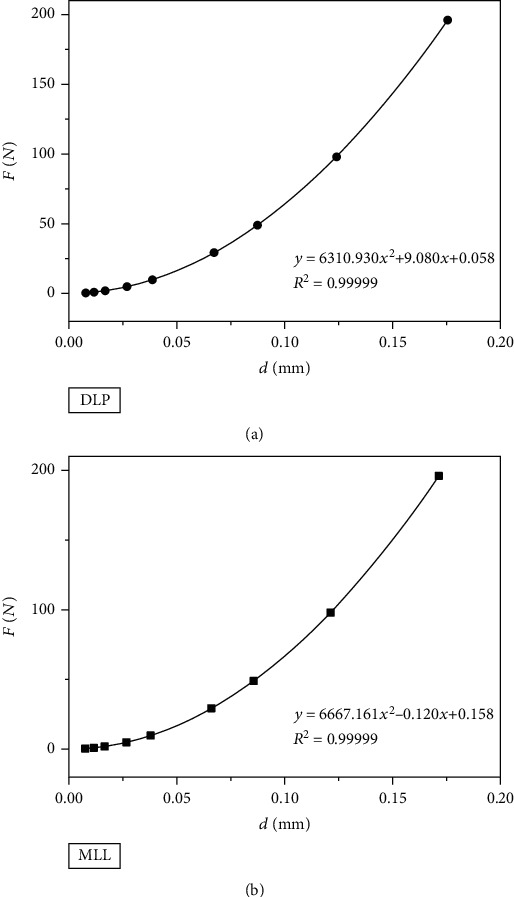
Fitting curves of *F* versus *d* based on the MPSR model. (a) DLP group. (b) MILL group.

Polynomial regression was applied to *F* versus *d* to obtain *a*_0_, *a*_1_, and *a*_2_ (see Figure 6), and the results are displayed in Table 5. The true hardness HV_T_ (HV) could be calculated by the following equation [15, 16]:

(6) $$\begin{array}{c} {\text{HV}}_{\text{T}}=\alpha ∙a_{2}. \end{array}$$

As is shown in Table 6, the true hardness was 1189 HV for the DLP group and 1248 HV for the MILL group based on the PSR model and was 1193 HV for the DLP group and 1261 HV for the MILL group based on the MPSR model.

### 3.4. Fracture Toughness

**Figure 7:**
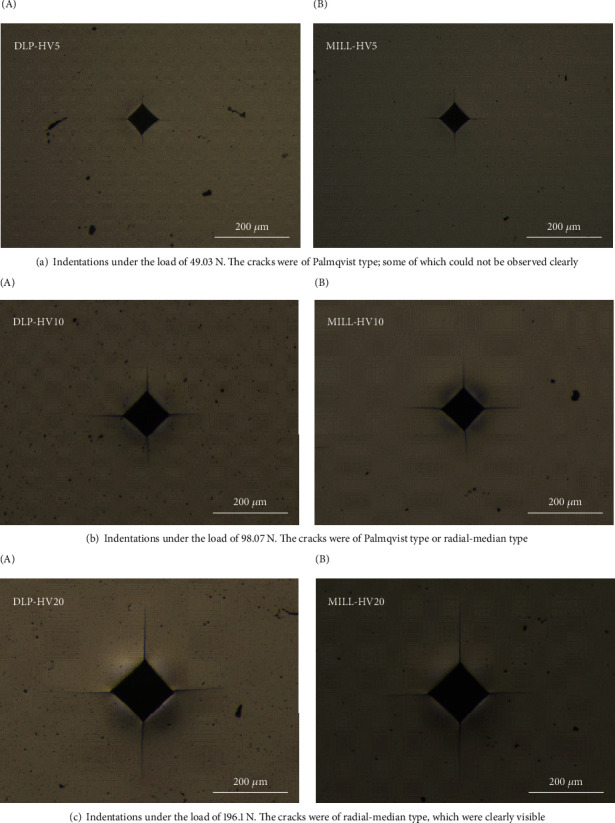
Optical micrographs of Vickers indentations with cracks emanating from the indentation corner under loads of 49.03 N (a), 98.07 N (b), and 196.1 N (c).

**Figure 8:**
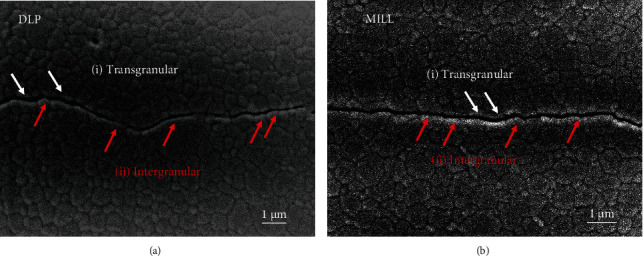
Indentation crack profiles. (a) DLP group. (b) MILL group. The cracks of both groups showed a mixture fracture mode of intergranular fracture (i) and transgranular fracture (ii).

According to the measurements of crack length *c* (mm) and half of the indentation diagonal *a* (mm), the *c*/*a* ratio was calculated to be smaller than 2.5 under the load of 49.03 N and larger than 2.5 under the load of 196.1 N, while under the load of 98.07 N, the *c*/*a* ratio showed a mixture type (see Figure 7). The fracture toughness value was calculated under the load of 196.1 N since it could generate clearly visible cracks of radial-median type, whereas the Palmqvist cracks noted at 49.03 N were not that clear. Therefore, the model developed by Anstis et al. [17] for the radial-median crack type was selected for the calculation of fracture toughness *K*_IC_ (MPa√m):

(7) $$\begin{array}{c} K_{\text{IC}}=0.016\times \frac{F}{c^{1.5}}\times {\left(\frac{E}{\text{HV}}\right)}^{0.5}, \end{array}$$

where HV = 1.8544 × (*F*/(2*a*)^2^) (MPa), *c* is the crack length from the indentation center to the crack tip (mm), and *E* is Young's modulus of zirconia (GPa). The fracture toughness value was 3.43 ± 0.29 MPa√m for the DLP group and 3.44 ± 0.23 MPa√m for the MILL group. Student's *t*-test was conducted using the SPSS 22.0 software (IBM, Armonk, USA) to analyze the difference in *K*_IC_ values between the two groups. The results indicated that there was no significant difference between DLP-manufactured zirconia and milled zirconia (*P* > 0.05). The indentation crack profiles of both groups are shown in Figure 8. Cracks of both groups presented a mixture fracture mode of intergranular mode in the predominant role and transgranular mode.

## 4. Discussion

The results of this study show that the Vickers hardness of DLP-manufactured zirconia was statistically lower than that of milled zirconia while there was no statistical difference in fracture toughness between two groups of zirconia, thus rejecting the null hypothesis.

In this study, the dental zirconia specimens were manufactured by two techniques. Since the 3D printing zirconia slurry used in this study was 3 mol% yttria partially stabilized tetragonal zirconia polycrystal (3Y-TZP), which is composed of similar contents to the commercial nontransparent zirconia (Table 1), the Zenostar MO presintered Y-TZP discs were chosen as the material of the control group. Besides the similar composition contents, the same final sintering procedure was selected to densify the parts of both groups. Thus, the main difference was in the forming of the parts before the final sintering. As for the numerically controlled milling technique, the zirconia green body is shaped by isostatic cold pressing, followed by a presintering procedure to achieve a certain strength [18]. Regarding the DLP 3D printing technique, the green body is printed by curing the photosensitive ceramic slurry through light projection; then, the debinding process is carried out to remove the organic parts [19]. The results of microstructural characterization showed that the density, grain size, and phase composition of the two groups were similar to each other, indicating that the difference in forming the parts before the final sintering almost had no influence on the microstructure of zirconia. To some extent, it can be predicted that the mechanical properties including hardness and fracture toughness of zirconia manufactured by the two techniques would be close [20].

The hardness and fracture toughness of the DLP-manufactured zirconia were determined by the indentation technique, which is a standard method for determining the Vickers hardness [10] while not the recommended method for testing fracture toughness [21]. However, the indentation fracture method is a rather popular technique due to its ease and convenience of operation since the *K*_IC_ values could easily be calculated according to empirical formulas based on the results of Vickers indentation tests. Although the *K*_IC_ values determined by the indentation fracture method could just be used as approximations of true fracture toughness values, they were good indicators for comparing the fracture toughness between two materials.

**Figure 9:**
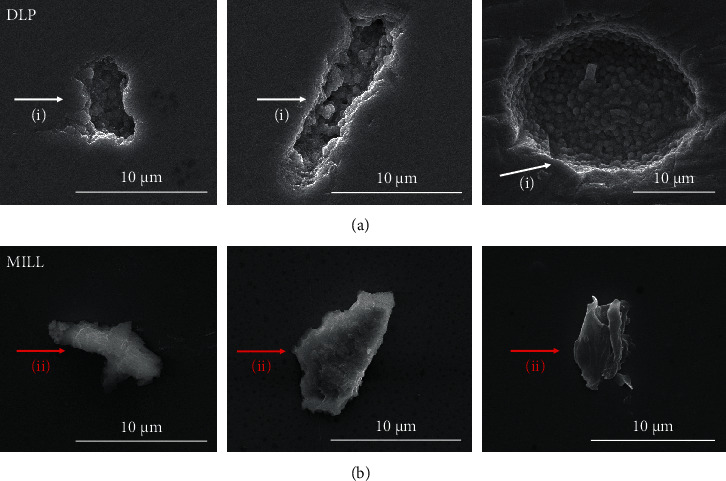
SEM images of polished surfaces. (a) DLP group with (i) pores of diverse shapes and sizes. (b) MILL group with (ii) aggregates of uniform sizes.

The hardness was tested under a range of loads from 0.49 N to 196.1 N. The results revealed that both groups of zirconia had normal ISE, which was further verified by Meyer's law, indicating that the obtained results of hardness were apparent hardness dependent on the applied loads. The apparent hardness of DLP zirconia was a bit statistically lower than that of MILL zirconia for 2% at 0.49 N, 4% at 0.98 N, 3% at 1.96 N, 1% at 4.9 N, 4% at 9.81 N, 4% at 29.42 N, 4% at 49.03 N, 4% at 98.07 N, and 5% at 196.1 N. The PSR model and MPSR model were applied to obtain the true hardness, and the results showed that the true hardness of DLP zirconia was 5% lower than that of MILL zirconia. Research has shown that the Vickers hardness has a strong dependence on porosity and pore size [22]. The hardness would decrease with increasing porosity and pore size. From the SEM images of polished surfaces of zirconia (see Figure 9), pores with diverse shapes and sizes could be observed in the DLP group, while agglomerates with uniform sizes were observed in the MILL group. For the DLP group, the pores tend to form during the coating process when the solid content of the slurry is too high, which leads to high viscosity [23]. And the forming of agglomerates is mostly due to the unstable suspension containing irregular aggregates of particles [24]. With regard to the MILL group, the formation of pores and aggregates may be related to the quality of the raw material powder. The study has also shown that the pores are often concentrated near the surface of the additive manufactured zirconia produced by horizontal building, and such defects on the surface of the tensile side would easily become the areas of stress concentration under the applied load, resulting in the failure of the specimens [25]. Although the relative densities of both groups were close to each other, the pores of the DLP group were mainly concentrated near the surface, while those of the MILL group were distributed throughout the entire volume. Therefore, the pores with large sizes on the surface of DLP zirconia might lead to its slightly lower Vickers hardness value.

Zirconia stands out from other ceramics mainly due to its high fracture toughness, which is an important property for the clinical use of bridge frameworks, especially those with large spans. It differs from other brittle ceramic materials in that it would just generate clear indentations with cracks under large loads like metal materials do without any fracture or chipping [26]. The toughening mechanism of zirconia is due to the stress-induced phase transformation. To be specific, when zirconia is subjected to a high load with fracture cracks emanated, the tetragonal phase stabilized at room temperature would transform to the monoclinic phase, causing 3%-5% of volume expansion, which generates compressive stress to prevent cracks from propagation. There was no statistical difference found in the fracture toughness values of both groups, and the main reason for that might be the highly similar microstructure characterization of the two groups of zirconia. As is known, the fracture toughness is mainly dependent on the grain size and grain boundary, which determine the energy required for the crack extension. The higher the energy is required, the higher the fracture toughness is. The XRD pattern showed that DLP zirconia and MILL zirconia have the same phase composition, and the SEM images displayed that the grain arrangement of both groups was even and dense with similar size, thus explaining the results of fracture toughness measurements. However, the *K*_IC_ values of zirconia were a bit lower than those of previous research studies [27, 28], which may be attributed to the difference among the different empirical formulas. A study has shown that the fracture toughness value calculated by the Anstis model was relatively lower than that calculated by other empirical models [11]. Although the indentation fracture method could not be used as a standard method or quantitative determination for the evaluation of ceramic fracture toughness [29], it is a relatively easy and effective method for comparing two materials.

Based on the previous research on the flexural strength of DLP-manufactured Y-TZP [7], this study further investigated its hardness and fracture toughness to achieve a comprehensive understanding of the basic mechanical properties compared to commercial milled Y-TZP. The limitation of this study is that the indentation fracture method used to determine the fracture toughness is not the standard method recommended in the ISO 6872 [21]. The *K*_IC_ values calculated by empirical formulas relying on indentation crack lengths could not be used as the true values of fracture toughness. Meanwhile, the dynamic mechanical properties like fatigue property and degeneration behavior need to be studied, and the biocompatibility, printing accuracy, and aesthetic performance also deserve exploration to provide an all-rounded theoretical basis for the clinical application of DLP-manufactured dental Y-TZP.

## 5. Conclusion

According to the study above, the following conclusions can be drawn:

1. The DLP-manufactured zirconia is similar to milled zirconia in microstructure, including density, grain size, and crystalline phase composition
2. The true hardness of DLP-manufactured zirconia is 5% lower than that of milled zirconia, which may be attributed to the existence of large pores on the surface of DLP zirconia
3. There is no statistical difference in fracture toughness between DLP-manufactured zirconia and milled zirconia, which may be due to the highly similar microstructure of both groups of zirconia
4. DLP-manufactured dental zirconia can achieve the requirements of dental prostheses in basic mechanical properties

## Figures and Tables

**Table 1:** Group names, components, and manufacturers of zirconia.

Group	Material	Components (mass%)	Manufacturer
DLP	Y-TZP slurry (solid content with 58 vol%)	ZrO_2_+HfO_2_+Y_2_O_3_ (99.72)	QuickDemos Company (China)
		Y_2_O_3_ (5.22)	
		Al_2_O_3_ (0.24)	
		Remaining (0.04)	
MILL	Presintered Y-TZP disc (Zenostar MO)	ZrO_2_+HfO_2_+Y_2_O_3_ (≥99.0)	Ivoclar Vivadent (Liechtenstein)
		Y_2_O_3_ (4.5~6.0)	
		Al_2_O_3_ (≤1.0)	
		Remaining (≤5.0)	

**Table 2:** The density, grain size, and crystalline phase structure of zirconia.

Group	Density (g/cm^3^)		Relative density (%)	Grain size (*μ*m)		Crystalline phase structure	Young's modulus (GPa)	
	Mean	SD		Mean	SD		Mean	SD
DLP	6.0198	0.0213	99.0099	0.6030	0.0326	Tetragonal	221.4	2.2
MILL	6.0382	0.0115	99.3125	0.5911	0.0330	Tetragonal	225.1	3.4

**Table 3:** The fitting results of Meyer's law.

Group	*n*	ln *K*	*K* (N mm^-n^)	*R* ^2^
DLP	1.932 ± 0.013	8.603 ± 0.044	5447.979	0.99961
MILL	1.935 ± 0.016	8.650 ± 0.056	5710.147	0.99942

**Table 4:** The fitting results of the PSR model.

Group	*a*_1_ (N mm^−1^)	*a*_2_ (N mm^−2^)	*R* ^2^
DLP	12.402 ± 0.823	6289.256 ± 9.995	0.99998
MILL	9.881 ± 1.513	6598.320 ± 18.783	0.99994

**Table 5:** The fitting results of the MPSR model.

Group	*a*_0_ (N)	*a*_1_ (N mm^−1^)	*a*_2_ (N mm^−2^)	*R* ^2^
DLP	0.058 ± 0.081	9.080 ± 2.630	6310.930 ± 14.719	0.99999
MILL	0.158 ± 0.105	−0.120 ± 3.486	6667.161 ± 19.962	0.99999

**Table 6:** True Vickers hardness of the two groups of zirconia.

Group	PSR (HV)	MPSR (HV)
DLP	1189	1193
MILL	1248	1261

## Data Availability

The data used to support the findings of this study are available from the corresponding author upon request.
