# Corrigendum to “Determination of Hardness and Fracture Toughness of Y-TZP Manufactured by Digital Light Processing through the Indentation Technique”

**DOI:** 10.1155/2021/9796816

**Published:** 2021-04-24

**Authors:** Ziyu Mei, Yuqing Lu, Yuxin Lou, Ping Yu, Manlin Sun, Xin Tan, Junjing Zhang, Li Yue, Haiyang Yu

**Affiliations:** State Key Laboratory of Oral Diseases, National Clinical Research Center for Oral Diseases, West China Hospital of Stomatology, Sichuan University, Chengdu 610041, China

In the article titled “Determination of Hardness and Fracture Toughness of Y-TZP Manufactured by Digital Light Processing through the Indentation Technique” [1], incorrect images were used for Figures 1 and 2 due to an error during the preparation of the manuscript. Figures 1 and 2 should be corrected as follows:

## Figures and Tables

**Figure 1:**
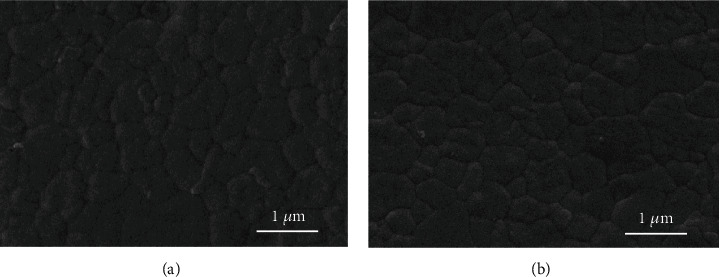
SEM images of the zirconia surface of the (a) DLP group and (b) MILL group at magnification ×40k and ×20k.

**Figure 2:**
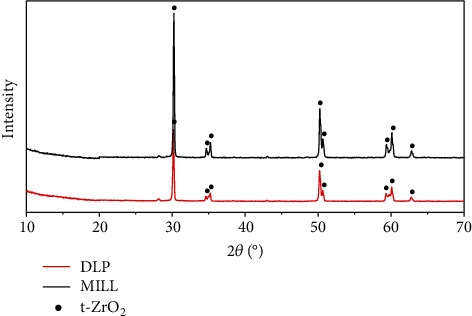
XRD patterns of zirconia.
